# Correlation Between Maternal-Fetus Interface and Placenta-Mediated Complications

**DOI:** 10.7759/cureus.62457

**Published:** 2024-06-16

**Authors:** Mihaela Andreescu

**Affiliations:** 1 Faculty of Medicine, Titu Maiorescu University, Bucharest, ROU; 2 Hematology, Colentina Clinical Hospital, Bucharest, ROU

**Keywords:** placental abruption, semi-allogeneic, placenta, maternal-fetus interface, decidua

## Abstract

Pregnancy is a highly regulated biological phenomenon that involves the development of a semi-allogeneic fetus inside the uterus of the mother. The maternal-fetal interface is a critical junction where communication takes place between the fetal and maternal immune systems, which determine the outcome of the pregnancy. The interface is composed of the decidua and placenta. The main cells present at the maternal-fetal interface include invading trophoblasts, maternal immune cells, and decidual stromal cells. Although maternal tolerance is crucial for maintaining a successful pregnancy, the role of the placenta in pregnancy is also important. Dysregulation of the placenta leads to various placenta-mediated complications, such as preeclampsia, intrauterine growth restriction, and placental abruption. Although the exact mechanism involving these complications is unclear, research has elucidated various factors involved in these pregnancy disorders. This review aimed to provide a summary of the maternal-fetal interface and immune mechanisms involved in placenta-mediated complications.

## Introduction and background

Pregnancy is a fine-tuned, highly regulated event that requires a balance between immune activation and embryonic antigen tolerance [1]. The successful outcome of pregnancy is dependent on maternal-fetal dialogue at various levels. As the fetus is semi-allogeneic, the maternal immune system should be able to show immune tolerance while also keeping defense intact against pathogens. Key predictors of pregnancy outcome include fetal immune tolerance, hormonal balance, and genetic and environmental factors [2]. The maternal-fetal interface, a dynamic and complex microenvironment, comprises decidua, uterine epithelium derived from the mother, and placenta-derived from the fetus [3]. The interface comprises various immune cells, such as T cells, B cells, natural killer (NK) cells, macrophages, and NKT cells [4].

The field of reproductive immunology has elucidated the pathophysiology of various gestational pathologies and complications. Normally, the human immune system works on the principle of self and non-self to identify pathogens. Upon presentation of non-self, it will trigger the immune response; however, in pregnancy, this hostile reaction is not manifested by the maternal immune system [5]. This behavior is caused by the recognition of paternal genes, with subsequent modifications in the maternal immune response to identify the fetus as a “temporary self” [6]. The maternal-fetal interface represents the direct point of contact between the mother and the developing fetus. The immune microenvironment is crucial for maintaining a healthy pregnancy. It involves the interplay of trophoblast cells, various immune cells such as NK cells, T lymphocytes, macrophages, and dendritic cells, along with numerous cytokines [7].

The placenta plays a vital role at the maternal-fetal interface. It controls vital physiological changes and is responsible for the nutrient supply to the fetus and its development. The placenta also restricts vertical transmission during pregnancy [8]. Several pregnancy complications occur due to abnormal placentation, leading to impaired perfusion of the placenta and, ultimately, placental dysfunction [9]. Common placenta-associated complications include preeclampsia, intrauterine growth restriction, and placental abruption. However, these complications are not that simple; they involve the interplay of multiple factors. This review article provides a summary of the maternal-fetal interface, decidua formation, placentation, and some placenta-mediated pregnancy complications.

## Review

Maternal-fetal interface

The maternal-fetal interface, also called the decidua, is the mucous membrane of the uterus during pregnancy [10]. It is immunologically tolerant toward genetically different fetuses while also maintaining a defense mechanism against potential pathogens. The deciduas form during early pregnancy. It originates from the differentiated endometrial cells and covers the placenta, umbilical cord, and fetus [11]. The decidua is composed of different parts based on its anatomical relationship with the blastocyst, parietalis, decidua basalis, and capsularis. The decidua basalis builds from the endometrium of the site of embryo implantation and encases the basal area of the placenta. The parietalis lines the membranes of the fetus, and the capsularis encases the uterine cavity parts [12]. The maternal-fetal interface plays a significant role in mediating the levels of nutrients, carbon dioxide, and oxygen [13]. It also generates various hormones, cytokines, and enzymes and exerts immunological protection to avoid any complications during pregnancy [4].

Decidua formation

Decidualization is the process of the formation of decidua and results in increased secretion of the uterine gland, alterations in the morphology of human endometrial stromal cells (hESC), reformation of blood vessels to provide nutrients and gases from the maternal blood to the embryo, and elevation of the number of uterine natural killer (uNK) cells [14,15]. The main cell type of the decidua becomes the decidualized hESCs [16]. During the formation of deciduas, hESCs differentiate into cells having an epithelial shape during the secretory phase [17]. Decidualized hESCs are characterized by the presence of large round nuclei, more nucleoli, the presence of accumulated glycogen and lipid in the cytoplasm, and the presence of dense secretory granules surrounding the boundary of the membrane [18]. The decidualization of the endometrium starts when the levels of progesterone increase before and after ovulation. The stromal cells present around the arteries induce morphological changes in the middle of the secretory period. Implantation is possible only six days post-ovulation. Decidualization is the first of all of the events of the complex pregnancy process [19].

Placentation

The development of the placenta during pregnancy occurs gradually and in a poorly understood manner. The embryo, after successive cell divisions, undergoes complicated interactions with the uterus as a blastocyst. Once the embryo is successfully implanted, it attaches itself to the endometrium and invades the maternal circulation and epithelium, starting the process of placentation [20]. The process of placenta formation occurs sequentially. During the first prenuclear stage, the mononucleated cells fuse to form the primary syncytium or the first syncytiotrophoblasts. The outer layer of the placenta is the syncytium and is in close contact with the maternal circulation. In the next stage, the lacunar stage, lacunae (fluid-filled spaces) appear within the primary syncytium. Trabeculae are the syncytiotrophoblasts that surround the lacunae. Two layers, named the basal layer and the superficial layer, coat the system of lacunae and trabecula [21]. Till the end of the first trimester, maternal circulation is poorly established in the placenta. A bilayer structure made of extraembryonic mesodermal cells and cytotrophoblasts appears on the 12th day after conception. This structure is named the chorion, the fetal portion of the placenta [22].

On day 15, chorionic cytotrophoblasts enter the syncytial mass of the trabecula, arrive at the maternal part of the placenta, and transform into interstitial trophoblasts and endovascular trophoblasts. Interstitial trophoblasts stay in the endometrial deciduas, while endovascular trophoblasts start to modify the spiral arteries and result in the replacement of the smooth muscle cells and the endothelium by trophoblasts. It results in the loss of elasticity of the vessels [23]. In the villous stage, the proliferation of the trophoblastic trabecula occurs, resulting in the formation of protrusions into the maternal blood circulation that surrounds the trabecula [24]. The extra-embryonic mesodermal cells from the chorionic plate enter the trabecula [25]. The extra-embryonic mesodermal cells enter the primary villi and transform them into secondary villi. Fetal capillaries appearing in the center of the secondary develop the tertiary villi. Additional branching and proliferation occur, leading to the formation of a system of villous trees. The blueprint of the placenta is formed in this way [26].

Immunology at the maternal-fetal interface

A successful pregnancy depends on the presence and proper function of maternal immune cells. During the first trimester, natural killer (NK) cells are the predominant type of maternal leukocytes present in deciduas. Other cells at this stage include T cells, dendritic cells, and macrophages [27]. A low number of other immune cells involving B cells, innate lymphoid cells, and mast cells are also present [28]. The number of natural killer cells remains stable during fetal development, but regulatory T cells expand with gestation and gather around trophoblasts [29]. An infection at the maternal-fetal interface poses a serious threat to the pregnancy. The placental trophoblast can detect and react to non-infectious agents and pathogens with the help of pattern recognition receptors (PRRs). This mechanism protects the placental immune system [30,31]. On the other hand, if these innate immune pathways are controlled inappropriately, various pregnancy complications associated with infections can occur by disturbing the phenotype, function, and normal distribution of the placental immune cells and placental function [27].

Maternal tolerance and pregnancy outcome

Maternal tolerance allows the mother to carry a genetically different fetus with foreign antigens. It is pivotal for a successful pregnancy outcome. Failure to develop maternal tolerance results in adverse pregnancy outcomes such as miscarriage and preeclampsia [32,33]. Tolerance is regulated by the modulation and control of leukocytes at the maternal-fetal interface. In decidua, the number of effector T class and dendritic cells (DCs) is low, while NK cells are present in abundance [34,35]. In the decidua, the differentiation of T cells into Treg cells occurs with the help of TGF-β and IL-10 cytokines. Another mechanism to gain immune privilege is apoptosis. Cytokines like TGF-β and IL-10 are secreted by macrophages in deciduas and play an essential role in promoting the growth of the fetus and preventing rejection until delivery [36].

Placenta-mediated pregnancy complications

Preeclampsia

Preeclampsia is reported in approximately 3% of pregnancies. It is a significant contributor to maternal and fetal mortality, with estimates suggesting 15% maternal mortality in developing countries every year [37]. Several etiological factors for preeclampsia have been reported; however, the exact pathophysiology is yet to be fully understood. There are multiple stages of preeclampsia, with the clinical manifestation of the disease being the last. Preeclampsia is characterized as a biphasic pathology wherein suboptimal placental perfusion elicits the secretion of specific factors, which leads to systemic vascular dysfunction, followed by clinical manifestations of the disease [38]. Preeclampsia can arise from both maternal and placental factors. Placental pre-eclampsia primarily arises from poor placentation during the early stages of pregnancy. This condition is characterized by the inadequate remodeling of the spiral arteries that furnish the uteroplacental circulation. Normally, placentation requires cytotrophoblasts to infiltrate the placental bed and establish contact with maternal tissues. Subsequently, the terminal portions of the spiral arteries undergo a process of dilation, resulting in expanded, homogeneous channels [39].

At the maternal-placental interface, maternal immune cells encounter various trophoblasts at different stages of pregnancy. These interfaces can be in decidua or maternal blood (intervillous space). In early-onset preeclampsia, uteroplacental perfusion is altered, which damages the syncytiotrophoblast. This leads to syncytiotrophoblast stress, resulting in syncytiotrophoblast fragment release, anti-angiogenic factors, and pro-inflammatory molecules [40]. These manifest as classical symptoms of preeclampsia. Therefore, syncytiotrophoblast stress signals are considered the most specific biomarker of preeclampsia [41]. Among other biomarkers, maternal blood has been analyzed for the levels of mRNA associated with both anti-angiogenic factors like vascular endothelial growth factor receptor-1 (Flt-1), endoglin (ENG), P-selectin, and placenta specific-1 (PLAC1), as well as pro-angiogenic factors such as placental growth factor (PlGF) and heme oxygenase-1 (HO-1) at the cellular level during fetal/placental development. A study by Sekizawa et al. showed that during the early second trimester, typically between 15- and 20 weeks gestation, a notable link exists between elevated mRNA expression levels of anti-angiogenic factors, including Flt-1, ENG, P-selectin, and PLAC1, and the subsequent onset of preeclampsia later in pregnancy. Additionally, women who develop preeclampsia exhibit significantly reduced mRNA levels of PlGF and HO-1 compared to those who do not develop preeclampsia [42]. However, a multi-center study by Widmer et al. reported that Flt-1, ENG, and PlGF are not effective during the early stages of pregnancy [43].

Uterine NK cells are the decidual immune cells that play a significant role in placentation. These cells are involved in the secretion of angiogenic mediators and cytokines during placentation, which helps in the remodeling of uterine spinal arteries. For this purpose, proper activation of these cells is required. These cells account for 70% of the total leukocytes during placentation. Any abnormality in the activation of uNK cells results in the development of preeclampsia [44]. A prospective study involving 30 pregnant women with preeclampsia and 20 healthy controls reported that there was a significant reduction in CD56+ NK cells (P<0.001) and an elevated number of CD68+ macrophages (P<0.001) compared to healthy controls [45]. Another study showed that women with preeclampsia had a high number of CD56+ and CD94+ cells. Their study further showed that pre-eclamptic women had lower levels of IL-12 in the placenta; however, this number was elevated in serum [46]. Wallace et al., in their study, used the uterine artery Doppler resistive index (RI) to diagnose pregnancies with high RI, which was indicative of impaired spiral artery remodeling [47]. Their findings showed that pregnancies with preeclampsia showed altered interactions with the trophoblast via decreased expression of HLA-binding cell-surface receptors. These can impact the transformation of the uterus for a successful pregnancy [47].

Intrauterine Growth Restriction

There is significant involvement of immune cells in the development and functioning of the placenta. Inadequate regulation of the placental immune environment is associated with pregnancy-related complications. Intrauterine growth restriction (IUGR) impacts around 5-10% of pregnancies [48]. In this condition, the fetus is unable to attain its prospective growth potential due to the harmful environment during pregnancy. It leads to a decrease in the growth velocity of the fetus after the first trimester. In the past, several studies have tried to uncover the molecular basis of IUGR. Elevated NOD1 protein levels contribute to the development of IUGR by raising inflammatory mediator levels. Additionally, increased leptin synthesis in placental trophoblast cells is linked to IUGR [49]. A study by Raghupathy et al. includes 36 women with IUGR and 22 normal controls in their analysis [50]. Their findings showed that IL-8, IFNγ, and TNFα levels were higher in the IUGR group compared to the control. Similarly, another study by Bezemer et al. reported that in IUGR patients, there appeared to be an increase in the inflammatory phenotype of decidual macrophages compared to healthy controls [51]. Similar findings have been reported from studies of preeclampsia and preterm birth, with elevated expression of the pro-inflammatory macrophage phenotype [52-55]. Sharps et al., in their study, also showed increased levels of placental macrophages along with inflammatory markers in the placenta in pregnancies in which the fetus had a reduced growth rate in the third trimester [56]. However, another study reported low levels of CD14+ macrophages in IUGR patients compared to healthy individuals [54]. Furthermore, increased levels of FOXP3+ Treg cells have also been reported in IUGR patients [51]. Some studies have also found a decrease in CD56+ uNK cells in IUGR pregnancies compared to healthy controls [54], whereas others did not find a significant difference [51].

Placental Abruption

Placental abruption (PA) is a relatively uncommon pregnancy complication, reported in approximately 0.4-1% of pregnancies [57]. PA is characterized by the detachment of the placenta before delivery. Although clinical manifestations of PA are often sudden, in the majority of cases, mortality occurs in utero without any symptoms. The exact mechanism of placental abruption remains unclear, though several risk factors have been identified. These include advanced maternal age, a previous occurrence of placental abruption, stimulant use, high blood pressure, and its associated conditions (such as preeclampsia or fetal growth restriction [FGR]), infections, polyhydramnios, preterm or prolonged rupture of membranes, a history of cesarean section, multiple pregnancies, and thrombophilia [58-60]. The immunological alterations in PA are not well documented in the literature. However, the disruption of NKs and T cell balance has been implicated in PA [61]. Research has shown that a heightened risk of placental abruption is associated with increased infiltration of macrophages and neutrophils in the uterus. Some studies have reported that placental abruption is notably linked to a buildup of cytotoxic responses due to the inadequate immunosuppressive function of the decidua [62,63].

Similarly, the role of inflammation has been reported in PA. A study by Nath et al. reported that chorioamnionitis was 18.3 times more common in PA compared to full-term pregnancy [64]. Proper placentation relies on the successful invasion of extravillous trophoblasts into the decidualized endometrium and the transformation of spiral arteries. Decidualization alters the expression of various factors in human endometrial stromal cells, such as tissue factor (TF), type-1 plasminogen activator inhibitor (PAI-1), and matrix metalloproteinases (MMPs), which play roles in maintaining hemostasis. Additionally, the decidua uniquely supports the coexistence of activated immune cells and decidual stromal cells. This environment regulates immune cell infiltration and activity, which is essential for immune tolerance during pregnancy and the viability of the fetus [65]. In PA, these molecular changes are terminated before uterine cervical ripening.

Prevention of adverse pregnancy outcomes

Interventions to avoid adverse pregnancy outcomes have not been very successful to date. Currently, no efficient intervention is present to prevent gestational diabetes, fetal growth restriction, stillbirth, and preterm birth [66]. Several studies have been conducted to explore preventive methods to avoid pregnancy complications. The use of aspirin is one of the suggested methods to decrease the risk of pregnancy complications [67]. A low dose of aspirin is recommended to prevent preeclampsia in patients. It is an inhibitor of the COX enzyme and reduces the production of pro-inflammatory mediators in the decidua. Hence, it promotes immune tolerance and facilitates the invasion of trophoblasts. However, the protective effect of aspirin is limited [66]. The use of low-molecular-weight heparin has also been studied to prevent pregnancy complications. It is one of the most commonly used methods but has limited supportive evidence [68].

## Conclusions

The development of a genetically different fetus inside the uterus is a complicated process. The maternal-fetal interface is responsible for the development of the embryo, and any complications at this interface result in adverse pregnancy outcomes. Commonly reported placental-related complications include preeclampsia, placental abruption, and intrauterine growth factor. Adequate preventive measures should be adopted to avoid these pregnancy complications and lower the risk of maternal and fetal mortality. These preventive methods have limited use and should be developed further.
